# mm-band surface acoustic wave devices utilizing two-dimensional boron nitride

**DOI:** 10.1038/s41598-022-24852-9

**Published:** 2022-11-29

**Authors:** Seok Hyun Yoon, Chang-Ki Baek, Byoung Don Kong

**Affiliations:** 1 Department of Electrical Engineering, Pohang University of Science and Technology (POSTECH), Pohang, 37673 Republic of Korea; 2 Department of Convergence IT Engineering, Pohang University of Science and Technology (POSTECH), Pohang, 37673 Republic of Korea

**Keywords:** Sensors, Electrical and electronic engineering, Actuators, Electronic devices

## Abstract

The simple structure, low power consumption, and small form factor have made surface acoustic wave (SAW) devices essential to mobile communication as RF filters. For instance, the latest 5G smartphones are equipped with almost 100 acoustic wave filters to select a specific frequency band and increase communication capacity. On the arrival of the newest communication standard, 5G, mm-band up to 39 GHz is supposed to be utilized, whereas the conventional SAW filters are limited to below 3 GHz, leaving a critical component missing. Here, we show an emerging 2D material—hexagonal boron nitride—can become a key enabler of mm-band SAW filter. Our study, based on first principles analysis and acousto-electric simulation, shows the operating frequency of SAW devices can reach over 20 GHz in its fundamental mode and 40 GHz in its interface mode with high electromechanical coupling coefficient (*K*^2^) and low insertion loss. In addition to the orders of magnitude improvement compared to the conventional SAW devices, our study provides a systematic approach to utilizing van der Waals crystals with highly anisotropic acoustic properties for practical applications.

## Introduction

Widely adopted in various electronic systems, including communications^1,2^, sensors^3–5^, and quantum information systems^6,7^, the surface acoustic wave (SAW) devices rely on the simple principle of the electromechanical resonances from the piezoelectricity^2^. SAWs on a piezoelectric material, induced by a set of interdigital transducer (IDT) electrodes with a period *λ*, propagate along the surface from one end, whereas another set of IDT electrodes, paired to detect the SAWs, convert the acoustic waves into the electric signal. Only signals around the resonance frequency can pass, and the paired IDTs serve as a bandpass filter. Their decisive advantage lies in the purely passive wireless readout capability embedded into a small form-factor^8^; they do not consume the battery. The fundamental resonance frequency (*f*_r_) is determined by *f*_r_ = *v*_SAW_/*λ*, where *v*_SAW_ is the speed of acoustic wave propagation at the surface, and this surface acoustic wave mode is called Rayleigh mode. LiNbO_3_^9–11^, LiTaO_3_^12^, ZnO^3–5,13–15^, AlN^16–19^, and AlScN^20,21^ are the piezoelectric materials commonly used or actively studied.

Ideally, the operating frequency could increase by reducing *λ*, but it cannot get shorter boundlessly. With nanoscale IDTs near the lithography limit, reliability issues arise due to the fabrication margins. As such, the operation frequency of the traditional SAW devices has been staggering under a few GHz. The harmonics of *f*_r_ (*f* = *n‧f*_r_, where *n* = 2, 3, 4, etc.) can provide resonances at higher frequencies, but significant insertion losses are the challenge. For instance, Zheng et al. recently reported a LiNbO_3_ SAW transducer with *λ* = 160 nm. The Rayleigh mode was 15 GHz, whereas a higher-order mode was 30 GHz (longitudinal bulk mode) with –40 dB insertion loss^11^. Büyükköse et al. demonstrated a 16.1 GHz ZnO SAW device with *λ* = 260 nm using the 4th order Rayleigh mode with -70 dB insertion loss^13^. Wang et al. showed a 33.7 GHz AlScN/diamond/Si multilayer SAW device with *λ* = 240 nm using the 3rd order Rayleigh mode with –20 dB insertion loss. In their work, the rare-earth Sc was to increase the electromechanical coupling, and the diamond substrate was to improve the sound velocity. They also reported a 17.7 GHz AlN/diamond/Si SAW device with *λ* = 500 nm and –20 dB insertion loss using Sezawa mode^17,20^—an interfacial higher-order mode between low and high sound velocity materials^22^.

A fundamental solution for a higher operating frequency is adopting a high *v*_SAW_ material, as it can be achieved with the same *λ*. Yet, the weak electromechanical coupling can be an issue. Usually, a material with high sound velocities, like a diamond, tends to be rigid since the acoustic wave speed is proportional to the crystal bonding strength. A rigid material requires more electric energy to induce the same mechanical variations, implying a low electromechanical coupling coefficient, *K*^2^^23^.

**Figure 1:**
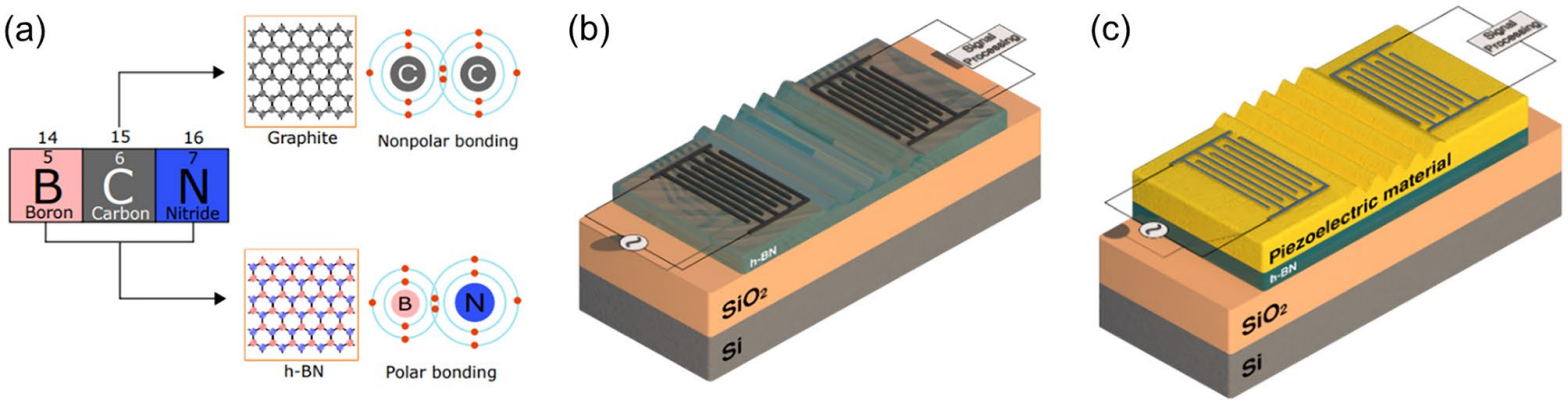
(**a**) Similarity of atomic structure of h-BN and graphite. h-BN has a polar bonding between two different elements, whereas those of graphite are non-polar. Schematics of SAW devices (**b**) utilizing h-BN as piezoelectric layer and (**c**) utilizing interfacial mode between piezoelectric material and h-BN.

This article presents mm-band SAW devices enabled by an emerging low-dimensional piezoelectric material, hexagonal boron nitride (h-BN). As a close cousin to graphene, h-BN has a very high *in-plane* sound velocity due to its strong covalent bonding between boron and nitrogen (Fig. 1a), which is similar to the strong carbon-to-carbon covalent bonding in graphene and diamond^24^. While graphene and diamond lack piezoelectricity due to their single elemental nature, h-BN maintains it along the *in-plane* direction^25^. As such, h-BN is uniquely positioned to provide a basis for a superior SAW device. For a practical RF filter beyond *Ka*-band (26.5–40 GHz), there are two possible approaches to utilizing h-BN. One is to excite the SAWs directly on h-BN, as illustrated in Fig. 1b, and the other is to adopt extra piezoelectric layers to stimulate the SAWs on h-BN, as shown in Fig. 1c. In the second case, the Sezawa mode is the primary target. The two approaches and their opportunities are explored in the following via acousto-electric simulations based on first principles analysis.

## Theoretical basis and calculations

### Piezoelectric properties of h-BN based on first principles

A good electromechanical coupling is essential for actuating and sensing the acoustic wave on h-BN. Although h-BN has been actively studied, the experimental and theoretical data from the literature were insufficient for the acousto-electric simulations. First principles analysis based on density functional theory (DFT) and density functional perturbation theory (DFPT)^26,27^ was adopted to obtain a complete set of parameters for the acousto-electric simulations. Quantum Espresso DFT suite was used with pseudopotentials and plane wave basis. Norm conserving local density approximation (LDA) with Perdew-Zunger (PZ) exchange correlation was adopted^28^. The electron energy cutoff was set up as 120 Ry with the first Brillouin zone sampling of 6 $\times$ 6 $\times$ 4 Monkhorst–Pack grids^29^. AA′ stacking of h-BN was assumed since it gives the energy minimum among three stable structures in the five possible stacking arrangements^30^. A recent experimental report on a high-quality single-crystalline multilayered h-BN (Ref.^31^) also supports that AA′ stacking is the most stable. The obtained equilibrium lattice constants *a* and *c* are 2.48 and 6.45 Å, respectively, showing excellent agreement with the experimental study. The piezoelectric properties were determined as differential quantities per geometric perturbation around the equilibrium position.

The highly anisotropic piezoelectric properties of h-BN are expected from the dissimilar bonding natures between the strong *in-plane* covalent bonds and the weak *out-of-plane* van der Waals forces^27^. This can be seen by the drastic difference between the *in-plane* and *out-of-plane* sound velocities. Extracted from the phonon dispersions, they are 19,600 m*/s* for *in-plane* and 3850 m*/s* for *out-of-plane*—*in-plane* is nearly five times higher. Our study reveals that this asymmetric acoustic propagation is a crucial factor in SAW devices utilizing van der Waals crystals. In a SAW device, the induced acoustic waves propagate at an effective velocity. This velocity is essentially an average of all possible propagation of elastic excitations within the film, including the multiple reflections at the interfaces. Unlike the bulk crystals, van der Waals crystals have an inherent large anisotropy in the acoustic wave velocities, and how those are averaged strongly influences device operations.

Using the DFT and DFPT, elastic stiffness constants (*C*_*ij*_) and optical dielectric constants were also calculated. *C*_*ij*_ are the response of a crystal to an externally applied crystallographic deformation where *i*, *j* = 1, 2, 3, 4, 5, and 6 (1 = *xx*, 2 = *yy*, 3 = *zz*, 4 = *yz*, 5 = *zx*, 6 = *xy*). *C*_*ij*_ was calculated from the energy-strain relation by adding four independent compressive and shear strains considering the crystal symmetry— ± 1% and ± 2% of deformation along the crystal axes^32^. The obtained elastic stiffness constants agree well with the theoretical and experimental results (Table 1)^24,33^. As expected, due to the weak van der Waals interactions, the components involved with the perpendicular axis to the basal plane are very small compared to those with the parallel axes. The macroscopic optical dielectric constants, parallel (${\varepsilon }_{\infty }^{\| }$) and perpendicular (${\varepsilon }_{\infty }^{\perp }$) to the basal plane, were obtained by Lyddane-Sachs-Teller relations (${\omega }_{\mathrm{LO}}^{2}/{\omega }_{\mathrm{TO}}^{2}={\varepsilon }_{0}/{\varepsilon }_{\infty }$) using the static dielectric constants (${\varepsilon }_{0}^{\| }$ and ${\varepsilon }_{0}^{\perp }$) and the optical phonon frequencies—longitudinal (ω_LO_) and transverse (ω_TO_)^34^. The calculated ${\varepsilon }_{\infty }^{\| }$ and ${\varepsilon }_{\infty }^{\perp }$ were 4.86 and 2.87, respectively. The *in-plane* direction has a higher value than the *out-of-plane* direction because the weak van der Waals interactions along the *out-of-plane* direction are less polarizable than the *in-plane* covalent bonding^35^. Finally, piezoelectric tensor elements, the quantities related to Born effective charge (${Z}^{*}$), were calculated as the derivatives of the forces (*F*) with respect to the electric field (*E*) created along the direction *α* and *β*(${Z}_{\alpha \beta }^{*}=\partial {F}_{\beta }/\partial {E}_{\alpha }$)^36^. All calculated quantities are compared favorably with the experimental and theoretical observations from the literature (Table 2)^33,35,37^.

**Table 1:** Elastic stiffness constants (*C*_*ij*_) of h-BN. (in GPa).

	*C*_11_	*C*_12_	*C*_13_	*C*_33_	*C*_44_	*C*_66_
Present work	928.78	211.26	2.58	31.98	17.83	358.76
Calc.^33^	951.5	169.2	2.5	28.2	–	–
Exp.^24^	811	169	0	27.05	7.7	321

**Table 2:** Dielectric properties of h-BN.

	${\varepsilon }_{\infty }^{\| }$	${\varepsilon }_{\infty }^{\perp }$	${\varepsilon }_{0}^{\| }$	${\varepsilon }_{0}^{\perp }$	$|{Z}_{\| }^{*}|$	$|{Z}_{\perp }^{*}|$
Present work	4.86	2.87	6.61	2.87	2.68	0.82
Calc.^33^	4.85	2.84	6.61	3.38	2.71	0.82
Calc.^35^	4.98	3.03	6.93	3.94	–	–
Exp.^37^	4.95	4.1	7.04	5.09	–	–

### Acousto-electric simulation

The acousto-electric simulation was performed based on the finite element method (FEM). Many studies have used this approach in SAW filter design, and the excellent predicting ability has been demonstrated^10–15,17,20,22^. The piezoelectric effects, the relations among stress (*T*), strain (*S*), electric field (*E*), and electric displacement field (*D*), are represented by the piezoelectric constitutive relations as^2^

1 $$T \, = \, c_{{\text{E}}} S \, - \, e^{{\text{T}}} E$$

2 $$D \, = \, eS \, + e_{0} e_{{{\text{rS}}}} E,$$

where *c*_E_, *e,* and *ε*_rS_ correspond to a material’s stiffness, coupling coefficient, and relative permittivity in their tensor form, respectively. *ε*_0_ is the vacuum permittivity. For the analysis of the SAWs, the nonlinear equations are solved using the finite element method, and COMSOL Multiphysics 6.0 was used for this study. Perfectly matched layers (PMLs) were applied at the boundaries unless they were periodic to prevent errors from the multiple reflections.

## Results and discussion

### h-BN based SAW bandpass filter

**Figure 2:**
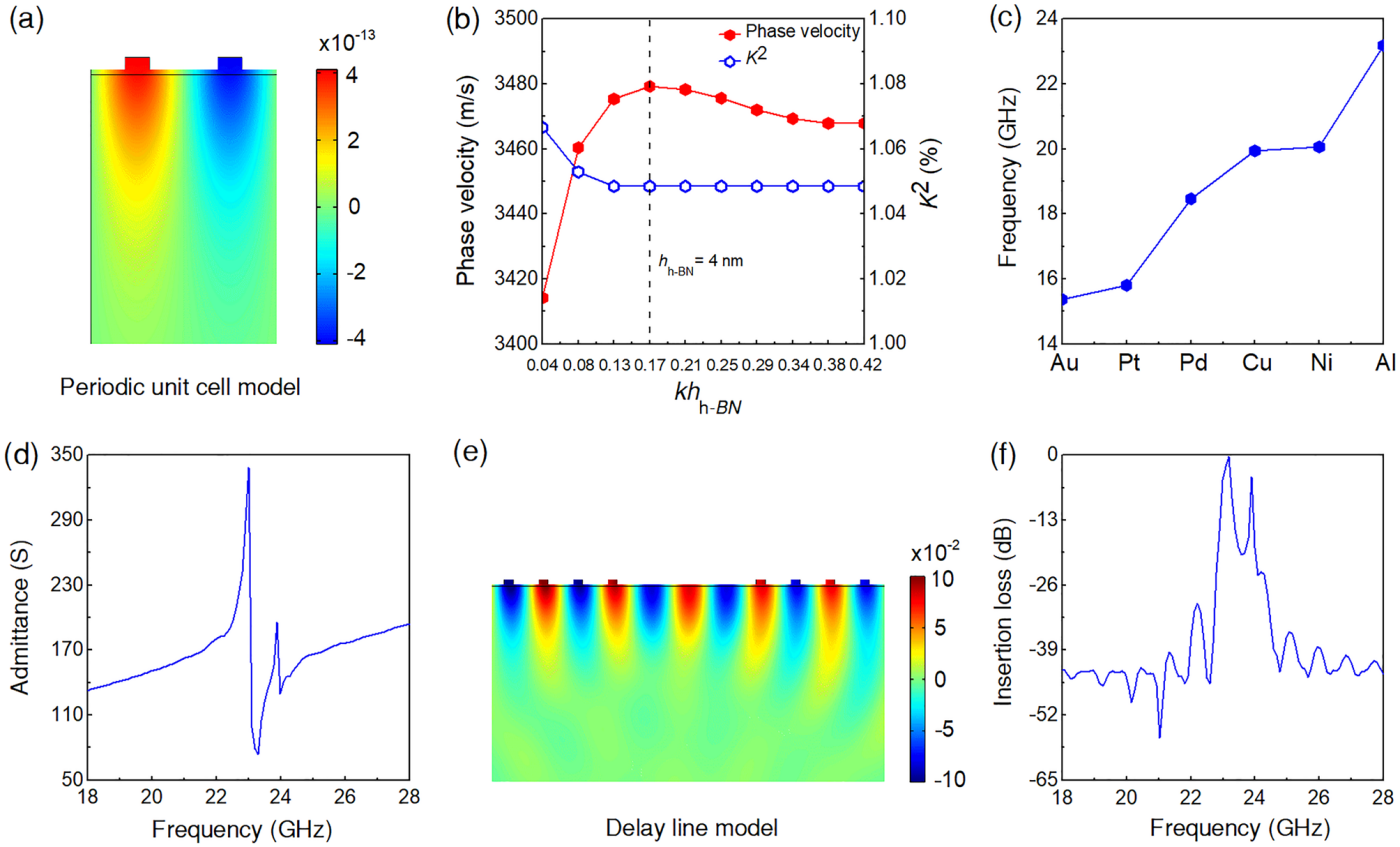
Frequency characteristics of h-BN/SiO_2_ layered structure. (**a**) Displacement field distribution (in nm) of a unit cell with 4 nm thick h-BN at Rayleigh mode frequency (23 GHz). (**b**) *kh*_h-BN_ dispersion curves of phase velocity and electromechanical coupling coefficient (*K*^2^). (**c**) Comparison of Rayleigh modes with different IDT metals. (**d**) Electrical input admittances vs. frequency near Rayleigh mode of delay line simulation with input and output IDTs. (**e**) Displacement field distribution of delay line simulation at Rayleigh mode. (**f**) Insertion loss (*S*_21_) of h-BN/SiO_2_ SAW filter from delay line simulation.

Using the properties from the DFT and DFPT calculations, the structure utilizing h-BN as a piezoelectric material (Fig. 1b) were investigated. The structure consists of h-BN, electrodes, and SiO_2_, which is a commonly used substrate for the h-BN transfer process^38^. For the periodic unit cell shown in Fig. 2a, eigenmode analysis was performed first. With conventional bulk crystals, a normalized thickness, *kh*, where *k* is the wave number and *h* is the thickness of the piezoelectric layer, is used to analyze the eigenmodes and phase velocities. However, the wave propagation velocity on an h-BN layer is a mixed value between the fast *in-plane* velocity and slow *out-of-plane* velocity, and is a function of *h*_h-BN_, the thickness of the h-BN film. To see the effect of the mixing of the asymmetric sound velocities by the thickness variation, the electrodes’ period (*λ* = 2π/*k*), thickness, and width were fixed as 150, 10, and 20 nm, respectively.

Figure 2b presents the *kh*_h-BN_ dispersion curves of the phase velocity and the electromechanical coupling. The electrode material is Al. There is an optimum *h*_h-BN_ to maximize the phase velocity and the electromechanical coupling. The electromechanical coupling (*K*^2^) can be determined using resonance frequencies (*f*_r_) and anti-resonance frequency (*f*_a_) from the input admittance curve^23^ using the relation given as

3 $$K^{2} = \frac{\pi }{2}\frac{{f_{r} }}{{f_{a} }}\tan \left( {\frac{\pi }{2}\frac{{f_{a}-f_{r} }}{{f_{a} }}} \right).$$

As the thickness decreases, the phase velocity increases because the fast *in-plane* contribution gets stronger. However, below 4 nm of *h*_h-BN_, the trend is reversed. The phase velocity decreases as the film gets thinner even though the fast *in-plane* portion increases. This is due to the loss of the piezoelectric volume. In the simulation, the height and width of the IDT electrodes, which are not piezoelectric but add total mass, were fixed. Thus, as *h*_h-BN_ decreases, the piezoelectric portion in the total volume decreases. Interestingly, *K*^2^ increases as *h*_h-BN_ becomes smaller than 4 nm, implying that the h-BN film becomes more piezoelectric. Yet, the loss of total piezoelectric volume dominates the trend, resulting in the reduced phase velocity. As such, 4 nm turned out to be the optimal thickness, with the highest phase velocity of 3470 m/s and *K*^2^ of 1.05%. The eigenfrequency of Rayleigh mode reached up to 23 GHz with Al electrodes. The displacement field distribution at the optimum case is shown in Fig. 2a.

Electrode materials and structures also affect the SAW generations. The trend is clear; the lighter the electrode, the higher the frequency due to the mass loading effect, which often significantly affects nanoscale acoustic devices^39^. Figure 2c compares the operation frequencies of Au, Pt, Pd, Cu, Ni, and Al electrodes with the same structure. Due to the large density, the lowest eigenfrequency with Au stayed at 15 GHz despite its high electrical conductivity − 35% less than the Al electrode. Naturally, the electrodes’ thickness is also of interest since it can influence the resonance frequency by changing the amount of the passive mass. Thus, the adopted IDT thickness was 10 nm of Al considering the electrical conductivity and the mass of metal electrodes. It is worth mentioning that a severe dependence on the electrode materials seems unusual in bulk piezoelectric crystals, and this is due to the weak mechanical strength of the h-BN layer.

To analyze the insertion loss of the h-BN/SiO_2_ structure, a delay line system with input and output IDTs was studied. The insertion loss is a major figure of merits of SAW filters since they are passive devices and represented by the scattering parameter (*S*-parameter) component *S*_21_. Each IDT comprised 25 pairs of electrode fingers. A small sinusoidal signal (amplitude 1 V) with frequency *f* was applied to the input IDT, and the electric signal at the output IDT was obtained. The input admittance curve (Fig. 2d) indicates the eigenfrequency at 23 GHz—almost identical to the result of the periodic cell study. From the displacement field distribution in Fig. 2e, the propagation of acoustic waves between the input and output IDTs can be clearly seen, and the concentrated acoustic energy near the top surface is the distinctive feature of the Rayleigh mode.

As shown in Fig. 2f, the insertion loss of only − 1 dB at 23 GHz is very promising. It should be noted that the structure is with only 25 pairs of IDT fingers, whereas actual SAW filters have a few hundred of them. The small number of IDT fingers was inevitable for the computational efficiency, but in an actual h-BN SAW implementation, even a lower insertion loss is expected by adopting more fingers. As such, the result suggests the h-BN/SiO_2_ SAW device has a solid potential to realize a high-performance high-frequency bandpass filter.

One issue revealed is the weak electromechanical driving force due to the thin film for high acoustic velocity. As the frequency goes above 25 GHz, the SAW device faces a significant insertion loss. Ironically, this degradation stems from the strong *in-plane* bonding and the high sound velocity—the very advantage of h-BN. The strong bonds appear as high vibrational energy and high sound velocity along the basal plane (steeper slopes in phonon dispersion). At the same time, this also means a rigid material; the required energy to excite enough elastic vibrations get higher. Adopting a faster sound velocity substrate, such as a diamond, can improve the resonance frequency. As well-known, a crystalline diamond has the highest sound velocity. In fact, in our previous study, a similar scale SAW device with h-BN on a diamond substrate showed an operating frequency above 44 GHz. However, *K*^2^ was still as low as 0.56% at the same time^40^. The AlN and AlScN SAW filters mentioned above also adopted diamond substrates to boost the resonance frequencies^17,20^. However, it may be very challenging to obtain a largescale crystal quality diamond and incorporate it into a mass fabrication process.

### Slow-on-fast multilayer structure for Sezawa modes

Multilayer SAW devices composed of h-BN and conventional piezoelectric materials possess another opportunity to take advantage of the fast *in-plane* sound velocity of h-BN. In this structure (Fig. 1c), the h-BN layer with high *in-plane* sound velocity provides the acoustic waves channel, while a bulk piezoelectric crystal with strong electromechanical coupling supplements the weak mechanical driving force of h-BN. In this case, the interfacial Sezawa mode from the *slow-on-fast* structure can support a resonance at a higher frequency than the fundamental Rayleigh mode^22^. This higher-order mode is essentially analogous to the surface electromagnetic waves generated in the total reflection at the interface between two media. As mentioned above, a diamond can play the same role, but the recent development of the wafer-scale crystalline growth of h-BN provides a clear advantage^31,41^.

**Figure 3:**
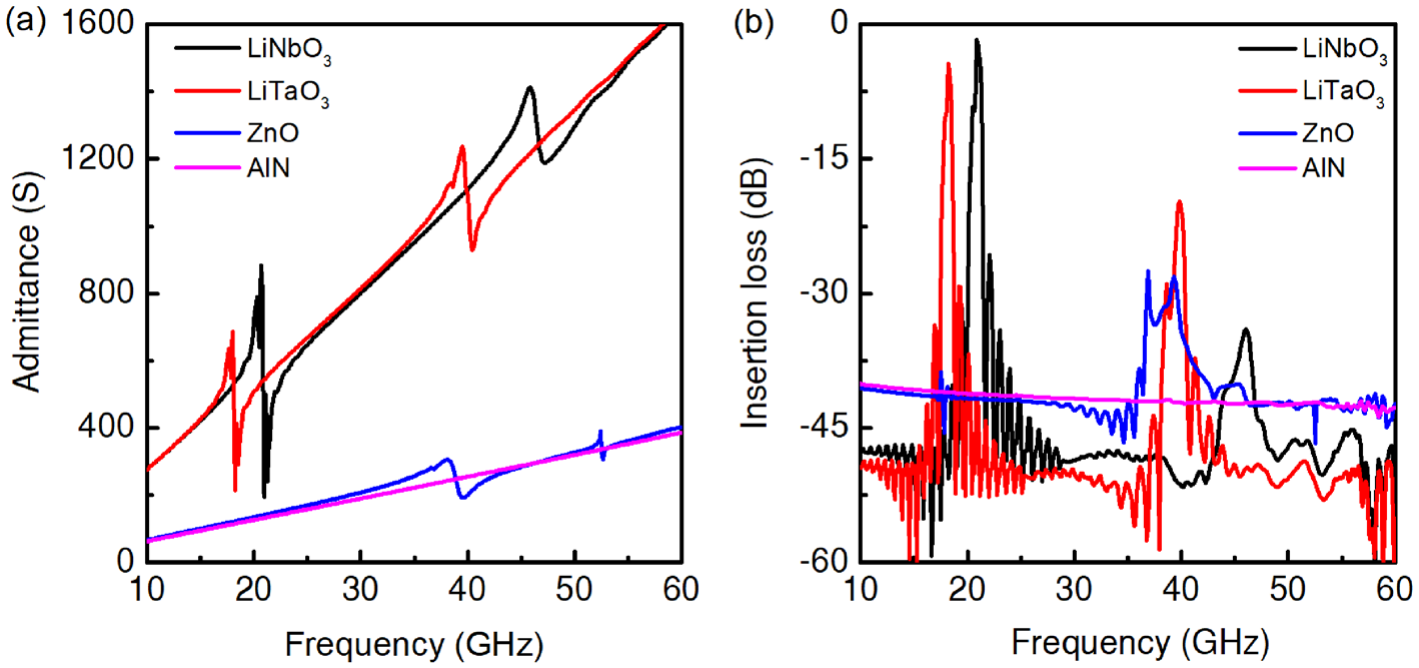
Frequency characteristics of piezoelectric material/h-BN/SiO_2_ SAW filters. (**a**) Electrical input admittance and (**b**) Insertion loss (*S*_21_) of the multilayer structures. 30 nm LiNbO_3_, LiTaO_3_, ZnO, and AlN are placed on h-BN/SiO_2_ (*h*_h-BN_ = 4 nm).

LiNbO_3_, LiTaO_3_, ZnO, and AlN are the conventional piezoelectric materials chosen to induce the Sezawa modes on h-BN. In this study, widely used *Y*-cut *X* propagating LiNbO_3_ and LiTaO_3_ (*Y-X* LiNbO_3_ and LiTaO_3_) are adopted due to their high SAW velocities and strong couplings^23,42^. Since the induced vibration near the IDTs should reach the interface with the underlying h-BN, the top layer’s thickness also affects the device’s performance. 30 nm of bulk piezoelectric layers turned out to be ideal for exciting the interfacial modes. The calculated input admittance and *S*_21_ with the same input and output IDTs are shown in Fig. 3. The key results are also summarized in Table 3. In the input admittance (Fig. 3a), the Sezawa modes (the second peaks) appear at higher frequencies than the Rayleigh modes (the first peaks) in all studied materials except for the AlN. The Sezawa modes easily reach up to 40 GHz or above with the same *λ* (150 nm). Considering the current lithography limits, there is room for further improvement if the IDT period is reduced. The results clearly demonstrate SAW filters for *Ka*-band (26.5–40 GHz) and beyond are possible by the adoption of h-BN as the fast acoustic channel and the judicious design of the multilayer structures. The best material for this is LiTaO_3_. The *S*_21_ of LiTaO_3_/h-BN/SiO_2_ structure was the lowest at –20 dB, with a 40 GHz operation frequency (Fig. 3b). LiNbO_3_ showed the highest operating frequency of 46 GHz, but a little more insertion loss of –28 dB. LiNbO_3_ and LiTaO_3_ are commonly used piezoelectric materials due to their high *K*^2^, but their operating frequencies have been under 1 GHz. The synergistic combination of the fast acoustic channel and the high electromechanical coupling enabled the SAW device for the extremely high frequency range.

**Table 3:** Comparison of operating frequency, phase velocity, insertion loss, and *K*^2^ at Rayleigh and Sezawa modes of multilayer structures.

Structure	Mode	*f*_r_ (GHz)	*v*_SAW_ (m/s)	Insertion loss (dB)	*K*^2^ (%)
LiNbO_3_/h-BN/SiO_2_	Rayleigh mode	21	3150	− 1.7	3.43
	Sezawa mode	46	6900	− 34	7
LiTaO_3_/h-BN/SiO_2_	Rayleigh mode	19	2850	− 4.7	2.64
	Sezawa mode	40	6000	− 20	5.3
ZnO/h-BN/SiO_2_	Rayleigh mode	17	2550	− 38.8	–
	Sezawa mode	39	5850	− 28.2	8.85
AlN/h-BN/SiO_2_	Rayleigh mode	–	–	–	–
	Sezawa mode	–	–	–	–

Regarding the insertion loss, low insertion loss (< –5 dB) h-BN SAW filters are possible up to 20 GHz by using either the Rayleigh modes or the Sezawa modes. However, some level of insertion loss is unavoidable in *Ka*-band. Since no reported SAW device is operating at this high frequency, a direct comparison is difficult. The previous studies demonstrated SAW filters under or near 30 GHz by Sezawa modes with the measured insertion loss ranges from −20 to −70 dB^11,13,17,20^. Besides, our study’s –20 dB insertion loss is with only 25 pairs of IDT fingers, but the real SAW filters usually have hundreds of IDT fingers. As mentioned above, the SAW design and simulation by the FEM showed good agreement in many studies, but implementing the real-scale device into the simulation is very difficult due to the computational limits. The usual techniques, such as acoustic reflectors, which can reduce the insertion loss, were not included in our simulation. In an actual device, the acoustic engineering with more IDT fingers may improve the performance further.

Among the investigated materials, ZnO demonstrated only Sezawa mode around 39 GHz without a clear sign of Rayleigh mode (Fig. 3b). A very weak resonance, however, can be found in the *S*_21_ chart (the wiggling around 17 GHz). This absence of Rayleigh mode is possibly due to the low *K*^2^. In fact, the weak coupling is not purely of ZnO but of the multiplayer structure and the acoustic impedance between ZnO and h-BN. In a separate simulation with only ZnO, the Rayleigh mode was clearly distinguished. Although the ZnO/h-BN structure showed the highest *K*^2^ of the Sezawa mode around 39 GHz, its transmission envelope turned out to be inappropriate as a bandpass filter. The insertion loss of ZnO/h-BN was also substantial, about –38.8 dB.

As for AlN, the studied structure displayed no resonance feature because of the low *K*^2^. AlN and h-BN are very rigid, so the applied input signal (peak-to-peak 1 V) seems insufficient to excite the acoustic waves. Since the signal from the antenna is expected to be at a low power level, the high required voltage for operation is unideal for an RF filter, which eliminates the material from the candidate list.

**Figure 4:**
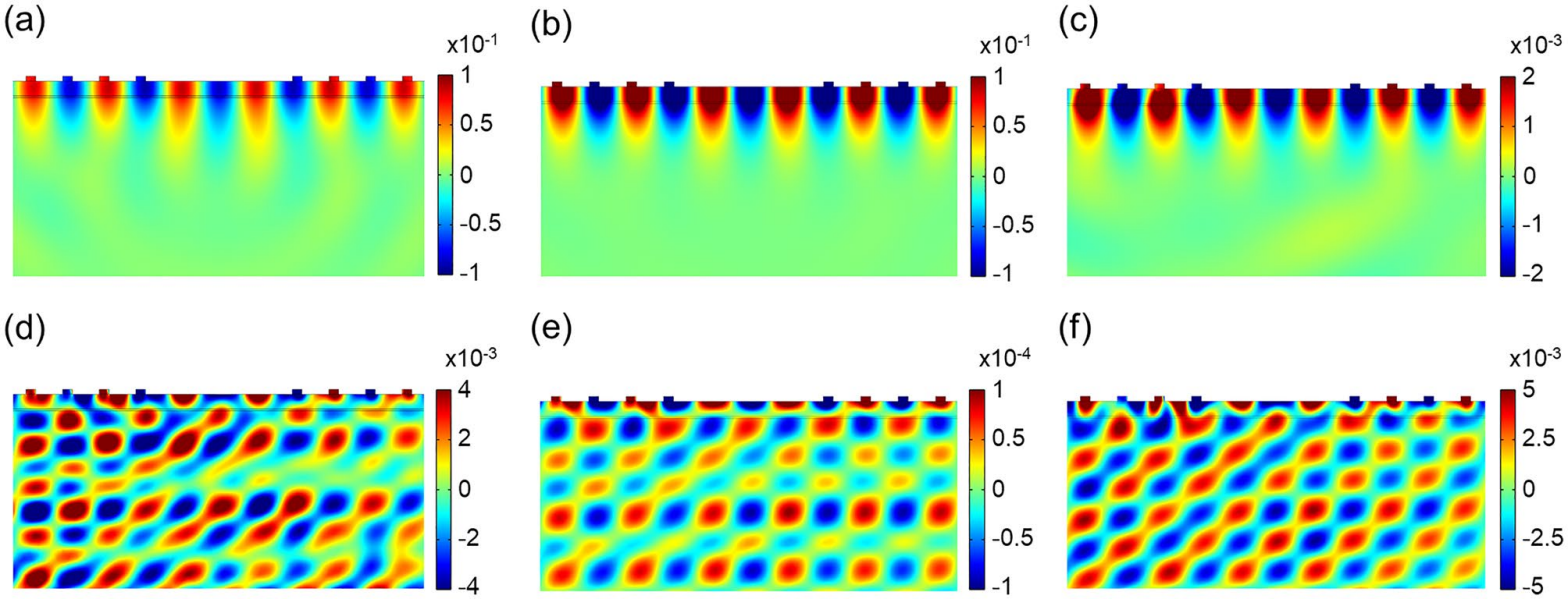
Distribution of displacement field (in nm) of piezoelectric material/h-BN/SiO_2_ delay line model. (**a**–**c**) Rayleigh mode with energy concentrated on the surface for LiNbO_3_/h-BN/SiO_2_, LiTaO_3_/h-BN/SiO_2_, and ZnO/h-BN/SiO_2_, respectively. (**d**–**f**) Sezawa mode with energy penetrated down to the interface and substrate for LiNbO_3_/h-BN/SiO_2_, LiTaO_3_/h-BN/SiO_2_, and ZnO/h-BN/SiO_2_, respectively.

The displacement field distributions of each device’s Rayleigh and Sezawa modes are shown in Fig. 4 (except for AlN since they are absent). The Rayleigh modes (Fig. 4a–c) show the acoustic energy localized near the top surface where the waves propagate. In contrast, the Sezawa modes (Fig. 4d–f) show the excitations penetrated down to the interface and even into substrate layers (h-BN and SiO_2_).

**Figure 5:**
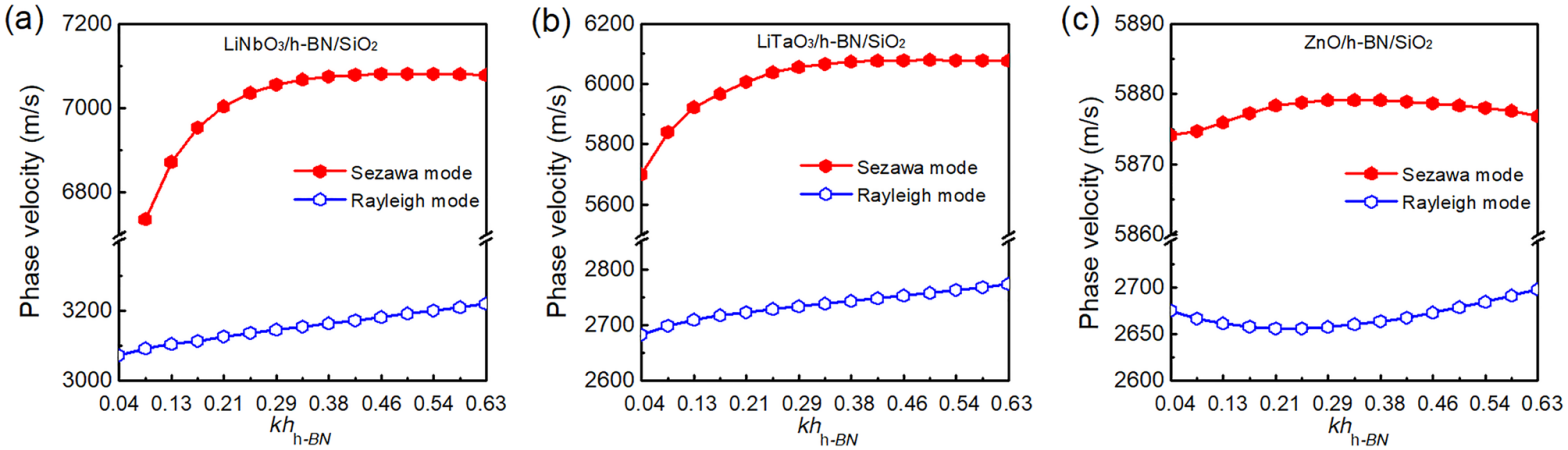
Characteristics of phase velocities depending on the *h*_h-BN_ with *kh*_h-BN_ dispersion curves (the *λ* is fixed at 150 nm). Phase velocities vs. h-BN thickness for Rayleigh and Sezawa modes of (**a**) LiNbO_3_/h-BN/SiO_2_, (**b**) LiTaO_3_/h-BN/SiO_2_, and (**c**) ZnO/h-BN/SiO_2_ multilayered structures.

The phase velocities of the multilayer structures were analyzed similarly; the *λ* is fixed at 150 nm, and only *h*_h-BN_ was changed. As shown in Fig. 5, the phase velocities of the Rayleigh modes increase in all structures as *h*_h-BN_ increases. However, the phase velocities of the Sezawa modes tend to saturate after a certain *h*_h-BN_. LiNbO_3_/h-BN and LiTaO_3_/h-BN show the maximum phase velocities of 7087 and 6079 m/s, respectively, with 12 nm of *h*_h-BN_, whereas ZnO/h-BN shows the maximum phase velocity of 5879 m/s with 7 nm of *h*_h-BN_. Beyond these thicknesses, the phase velocities either saturate or slightly decrease as the *in-plane* contribution of h-BN diminishes. In LiNbO_3_/h-BN, the higher-order modes disappear at *h*_h-BN_ = 1 nm due to the weak piezoelectric effects of too thin h-BN.

### Further improvement of h-BN SAW filters

Boron is a material known for its high isotope ratio. We adopted the natural ratio of boron isotopes (20% of ^10^B and 80% of ^11^B) in all our calculations. Nitrogen’s isotope rate is negligible (99.6% of ^14^N). This large portion of B isotopes works as mass defects in phonon transport due to the increased phonon scattering, which implies the fast decay of SAWs. By isotopically purifying B, the propagation of SAWs on h-BN can be improved, having insertion loss better. Actually, this isotope effect was observed experimentally in long optical phonon propagation (4 µm) on an isotopically purified h-BN film^43^. In addition, h-BN with ^11^B can have a slightly higher *in-plane* sound velocity since the binding energy of ^11^B is higher^44^. As such, isotopically purifying h-BN can further improve the operating frequency.

The h-BN growth technique is rapidly developing, and high-quality, single-crystalline wafer-scale h-BN has become obtainable recently^31,41^. In addition, h-BN is a van der Waals crystal that can be grown layer-by-layer. As such, the accurate thickness control is also an advantage^45,46^. Since the sputtering can be easily performed on any type of substrate, few difficulties are expected in fabricating the proposed multilayer structure. As for the IDTs, fabricating tens of nanometer scale metal lines is routine work with the current lithography technology^47^. For instance, Ref.^20^ demonstrated a SAW device of 30 nm IDT finger width, and the resolution limit of the EBL process is below 10 nm^48^. Thus, our study provides a convincing path toward a high-frequency SAW filter with an order of magnitude improvement from conventional devices.

## Conclusions

In summary, the SAW devices based on h-BN were studied based on the first principles analysis and acousto-electric simulations. We demonstrated that a SAW filter based on h-BN could become an unconventional promoter for the next-generation RF filter technology. The SAW devices adopting h-BN as a piezoelectric material can operate above 20 GHz with nearly zero insertion loss. The multilayer structure utilizing h-BN as a high-speed acoustic channel can operate even higher by its interfacial excitations. The strong electromechanical coupling, integrated into the high *in-plane* sound velocity, provides an operation beyond *Ka*-band (> 40 GHz) by the Sezawa mode, with a high *K*^2^ (> 5%) and considerably low insertion loss of − 20 dB. These are the highest operating frequencies ever researched by stacking conventional piezoelectric materials, including LiNbO_3_, LiTaO_3_, and ZnO. Thus, our result strongly suggests the exceptional properties of h-BN can be a swift channel toward the essential part of future communication technology.

## Data Availability

The data that support the plots within this paper and other findings of this study are available from the corresponding authors upon reasonable request.
